# Lessons From an Evaluation of a Training Program of Paid Eldercare Workers

**DOI:** 10.1093/geroni/igaa057.2497

**Published:** 2020-12-16

**Authors:** Liat Ayalon

**Affiliations:** Bar-Ilan University, Ramay Gan, HaMerkaz, Israel

## Abstract

The present study is based on a three-year evaluation of an Israeli training program for local paid elder care workers, called “community care.” Interviews were conducted with all stakeholders involved in the program, including program developers, facilitators, funders, trainees, dropouts, employers, and older care recipients. Qualitative thematic analysis was used, supplemented by quantitative data concerning the program’s inputs, outputs and outcomes. The program had multiple strengths, including a substantial funding stream and a highly skilled and committed team. Yet, out of 130 participants (in the 7 training programs evaluated), only 94 completed the program and 31 were later employed as care workers. Three main challenges to the efficacy of the training program were identified. The findings stress the importance of adequately conducting the appropriate needs assessment prior to embarking on a new social program and illustrate the tension between an ideal prototype and real-life constraints.

